# CRISPR-Cas12b enables a highly efficient attack on HIV proviral DNA in T cell cultures

**DOI:** 10.1016/j.biopha.2023.115046

**Published:** 2023-06-28

**Authors:** Minghui Fan, Yuanling Bao, Ben Berkhout, Elena Herrera-Carrillo

**Affiliations:** Laboratory of Experimental Virology, Department of Medical Microbiology, Amsterdam UMC, Academic Medical Center, University of Amsterdam, Amsterdam, the Netherlands

**Keywords:** Antiviral, Lentiviral vector (LV), CRISPR-Cas12b, HIV cure

## Abstract

**Background:**

The novel endonuclease Cas12b was engineered for targeted genome editing in mammalian cells and is a promising tool for certain applications because of its small size, high sequence specificity and ability to generate relatively large deletions. We previously reported inhibition of the human immunodeficiency virus (HIV) in cell culture infections upon attack of the integrated viral DNA genome by spCas9 and Cas12a.

**Methods:**

We now tested the ability of the Cas12b endonuclease to suppress a spreading HIV infection in cell culture with anti-HIV gRNAs. Virus inhibition was tested in long-term HIV replication studies, which allowed us to test for viral escape and the potential for reaching a CURE of the infected T cells.

**Findings:**

We demonstrate that Cas12b can achieve complete HIV inactivation with only a single gRNA, a result for which Cas9 required two gRNAs. When the Cas12b system is programmed with two antiviral gRNAs, the overall anti-HIV potency is improved and more grossly mutated HIV proviruses are generated as a result of multiple cut-repair actions. Such “hypermutated” HIV proviruses are more likely to be defective due to mutation of multiple essential parts of the HIV genome. We report that the mutational profiles of the Cas9, Cas12a and Cas12b endonucleases differ significantly, which may have an impact on the level of virus inactivation. These combined results make Cas12b the preferred editing system for HIV-inactivation.

**Interpretation:**

These results provide in vitro “proof of concept’ for CRISPR-Cas12b mediated HIV-1 inactivation.

## 1. Introduction

Combination antiretroviral therapy (cART) is very efficient in virus suppression and has become the standard therapy for persons infected with HIV. Virus suppression can be maintained for many years, but only if drug therapy is continued [1,2]. Nevertheless, a cure is not realized by cART because the integrated HIV DNA genomes will persist in long-lived infected cells as a source for new rounds of virus spread. The Clustered Regularly Interspaced Short Palindromic Repeats/CRISPR-associated (CRISPR-Cas) system, which was discovered in 1987, has contributed much to basic research and already resulted in diverse applications, e.g. in the field of diagnostics, the construction of new model organisms and gene therapy [3–7]. CRISPR-Cas technology was developed such that targeted genomic lesions can be introduced with unprecedented efficiency, also making it a promising tool for HIV gene therapy [8–12]. The original Cas9 system and subsequently the Cas12a addition of the CRISPR-Cas type V family have been successfully applied as genome editing tools for mammals and other organisms [13–16].

More recently, the Cas12b member of the CRISPR-Cas V endonuclease family was identified, which was initially termed C2c1 [17,18]. This Cas12b endonuclease from *Bacillus hisashii* was developed by means of genetic engineering into an efficient genome editing tool for mammals. Cas12b shares several features with Cas12a, e.g. it recognizes a distal T-rich protospacer adjacent motif (PAM, which reads ATTN or TTTN), uses a seed region located at the PAM-proximal sequence that is five bases long [19], but it differs from Cas12a in generating sticky DNA breaks with a 7-nt 5′-overhang [17,18]. The Cas12b nuclease has a significantly smaller size (1108 amino acids or aa) than spCas9 and fnCas12a (1368 and 1307 aa, respectively), making it a more suitable tool for gene therapy applications that use a viral delivery system with a limited packaging capacity. Cas12b may be particularly attractive for HIV genome inactivation because it generates relatively large deletions [18] and because it is very sensitive to mismatches between the guide RNA (gRNA) and target DNA, thus causing less off-target effects than Cas9 and Cas12a and possibly presenting a more safe system for future clinical applications [18,20].

In previous studies, we and others exploited CRISPR-Cas strategies to target the HIV proviral DNA [21–25]. We reported effective HIV inhibition by Cas9 in infected cell cultures, documented viral escape when a single gRNA was used and complete inactivation of all infectious virus with certain combinations of two gRNAs [26,27]. Although an impressive result, the large size of Cas9 transgene cassettes impedes implementation in gene therapy applications with viral vectors that have a limited capacity to package transgene cargo, the “packaging capacity” [28]. We recently reported that Cas12a outperforms the original Cas9 system for antiviral activity as it was able to fully inactivate HIV with only a single gRNA [22]. The new Cas12b system has been studied less intensively and its ability to inhibit HIV was not probed yet. In this study, we demonstrate that Cas12b can efficiently block HIV replication and cure HIV infected cells with a single gRNA. We measured improved HIV-inactivation with two gRNAs and studied the molecular mechanism of HIV inactivation. CRISPR-edited HIV genomes with indel mutations at both Cas12b-target sites were prominent, but real excision events that remove the intervening sequences were rare. We conclude that Cas12b is a top candidate editing system for HIV cure strategies.

## 2. Materials and methods

### 2.1. Plasmid construction

The lentiviral plasmids PX458 (Addgene#62988) that harbors the spCas9 gene and a gRNA expression cassette, pY109 (LentiCpf1, Addgene # 84740) that harbors the LbCas12a gene and a gRNA expression cassette and the plasmid encoding BhCas12b variant 4 protein (Addgene #122446) were obtained from Feng Zhang’s laboratory [18,29]. A 3′-terminal hepatitis delta virus (HDV) ribozyme was introduced in the BhCas12b gRNA expression cassette to facilitate precise gRNA processing [30]. A gBlock gene fragment from Integrated DNA Technology (IDT) encoding the U6 promoter-gRNA scaffold-HDV cassette was inserted into the *Xho*I and *Pac*I restriction enzyme sites of pY109 to create plasmid pY109-HDV. This plasmid also contains two BsmBI sites for cloning of the anti-HIV spacer sequences. Subsequently, the EF1-α promoter and the BhCas12b v4 gene were cloned into the *Xho*I and *Bam*HI sites. To introduce a second gRNA, the gRNA expression cassette of pLenti-SpBsmBI-sgRNA-Hygro (Addgene plasmid # 62205) was PCR-amplified and cloned into the *Bam*HI and *Hpa*I sites [31]. The puromycin resistance gene was exchanged with the eGFP reporter gene by Gibson assembly according to the manufactures’ instructions (New England Biolabs) to determine the transduction titer of the LV stock. The plasmid pLAI encodes the primary CXCR4-using HIV isolate LAI (subtype B). The gRNA targets with top specificity score within highly conserved HIV sequences were prioritized (Supplementary Table S1).

### 2.2. Cell culture, transfection and transduction

Human embryonic kidney (HEK) 293 T cells were grown in DMEM (Life Technologies, Invitrogen, Carlsbad, CA, USA) supplemented with 10% fetal calf serum (FCS), penicillin (100 U/ml) and streptomycin (100 mg/ml) and cultured in an incubator at 37°C and 5% CO_2_. SupT1 T cells (ATCC CRL-1942) were cultured in advanced RPMI (GIBCO BRL, Carlsbad, CA, USA) supplemented with 1% L-glutamine, 1% FCS, penicillin (100 U/ml) and streptomycin (100 mg/ml) at the same culturing conditions.

For transient Cas12b/gRNA activity assays, HEK293T cells (at ~80% confluence in a 12-well plate) were transfected with 300 ng of plasmids encoding both CRISPR-Cas components (Cas12b and gRNA), 4 ng of pRL and 150 ng pLAI using Lipofectamine 2000 according to the manufacturer’s instructions. Two days post-transfection, the culture supernatant was collected for CA-p24 ELISA to measure virus production.

Production of lentiviral vectors in HEK293T cells and subsequent transduction of SupT1 T cells was conducted as previously described [32]. Briefly, HEK293T cells were transfected with the lentiviral plasmid and three packaging plasmids pSYNGP, pRSV-rev and pVSV-g using Lipofectamine 2000 (Invitrogen). Two days post-transfection, the lentiviral vector containing supernatant was harvested and centrifuged at low speed, filtered (0.45 µm) and concentrated 20-fold using Vivaspin 100.000 MWCO centrifugal filter units (Sartorius, Goettingen, Germany). SupT1 cells (2 × 10^5^ cells in 1 ml of culture medium) were transduced with the concentrated lentiviral vectors. After transduction, the cells were cultured in the presence of puromycin (1 µg/ml) for 7 days to select SupT1 cells expressing Cas12b and gRNA1, or hygromycin (500 µg/ml) for 14 days to select cells expressing gRNA2. The transduction titer of the Cas12b-eGFP and spCas9-GFP not concentrated and concentrated LV stocks was determined on the SupT1 T cell line by measuring eGFP transgene expression of transduced cells by fluorescence-activated cell sorting (FACS). Transduction titer was calculated following the formula: Transducing units per ml (TU/ml)= cell number x ((percentage of fluorescent cells/100)/dilution factor of the vector). The CA-p24 protein level was measured before and after concentration of the LV stock by an in-house CA-p24 ELISA assay.

### 2.3. HIV production and infection

To produce HIV LAI, HEK293T cells were transfected with 5 µg pLAI using Lipofectamine 2000. At two days post-transfection, the culture supernatant was collected, filtered (0.45 µm) and distributed in aliquots. The MOI was determined as previously described [33]. An equal amount of non-transduced SupT1 cells and transduced SupT1 cells (2 × 10^5^ cells in 1 ml medium) were infected with HIV (MOI 0.06 and 0.3) in six parallel cultures per each gRNA(s). The infected cells were cultured for 60 days and passaged twice a week. Virus replication was evaluated by scoring syncytia formation when cells were passaged.

To analyze candidate escape viruses, the culture supernatant was passaged onto fresh gRNA-transduced SupT1 cells to confirm the escape phenotype. Total cellular DNA (with integrated HIV proviruses) was isolated at the peak of the secondary infection with the QIAGEN DNAeasy kit and worked up for sequencing (see below). For cured cultures that did not demonstrate breakthrough virus replication, we first confirmed the absence of any replication-competent virus by mixing a culture sample with an equal number of control (non-transduced) SupT1 cells, followed by culturing for two weeks to confirm the absence of virus-induced syncytia. If a cure was confirmed, the gRNA targets were amplified by PCR (primers listed in Supplementary Table S2). The PCR program was optimized in an annealing temperature gradient (from 52°C to 61°C) using DreamTaq DNA polymerase (Thermo Fisher Scientific). The most specific PCR products were gel-purified and population-sequenced to confirm the presence of proviral DNA, then they were cloned in the TA-cloning vector (Thermo Fisher Scientific) and multiple TA-cloned fragments were analyzed by Sanger sequencing.

### 2.4. T7 endonuclease I (T7EI) cleavage assay

To detect gene editing induced by CRISPR-Cas12b, we performed T7E1 assays on extracted DNA from infected gTat1-transduced. The PCR product, which included both the on-target Tat1 site and potential off-target sites, was annealed and digested with T7EI using the EnGen Mutation Detection Kit (BioLabs). The NHEJ-induced DNA aberrations can be detected by gel electrophoresis, where the presence of smaller fragments indicates the occurrence of genome editing. We used plasmid HIV DNA treated with CRISPR-Cas12b-gCtrl and uninfected gCtrl-transduced cells as negative controls.

## 3. Results

### 3.1. Targeting HIV DNA with the Cas12bendonuclease and a single gRNA

The Cas12b endonuclease recognizes a distal T-rich PAM sequence (TTTN or ATTN) 1–4 bp upstream of the 23-nt spacer in the target DNA [34]. Cas12b activity is most robust on targets with ATTN as PAM than TTTN, reaching a level comparable to that of the original Cas9 system and the Cas12a endonuclease [18]. We tested the ability of Cas12b to inactivate the HIV by selecting targets on HIV genome with one of the two possible PAM motifs. As the Cas12a and Cas12b endonucleases share the same TTTN sequence as PAM, this allowed us to directly compare the antiviral activity of these editing systems on identical HIV targets. To do so, we choose four robust anti-HIV gRNAs from our previous Cas12a study that were selected against highly conserved regions of the HIV DNA genome [22] and tested their ability to trigger inactivation of the HIV DNA genome, but now by Cas12b. Disappointingly, Cas12b demonstrated very little to no HIV inhibition as virus replicated unhindered in the gRNA-expressing T cells (results not shown), indicating that Cas12b has poor antiviral activity, at least on targets with TTTN as PAM.

**Fig. 1.**
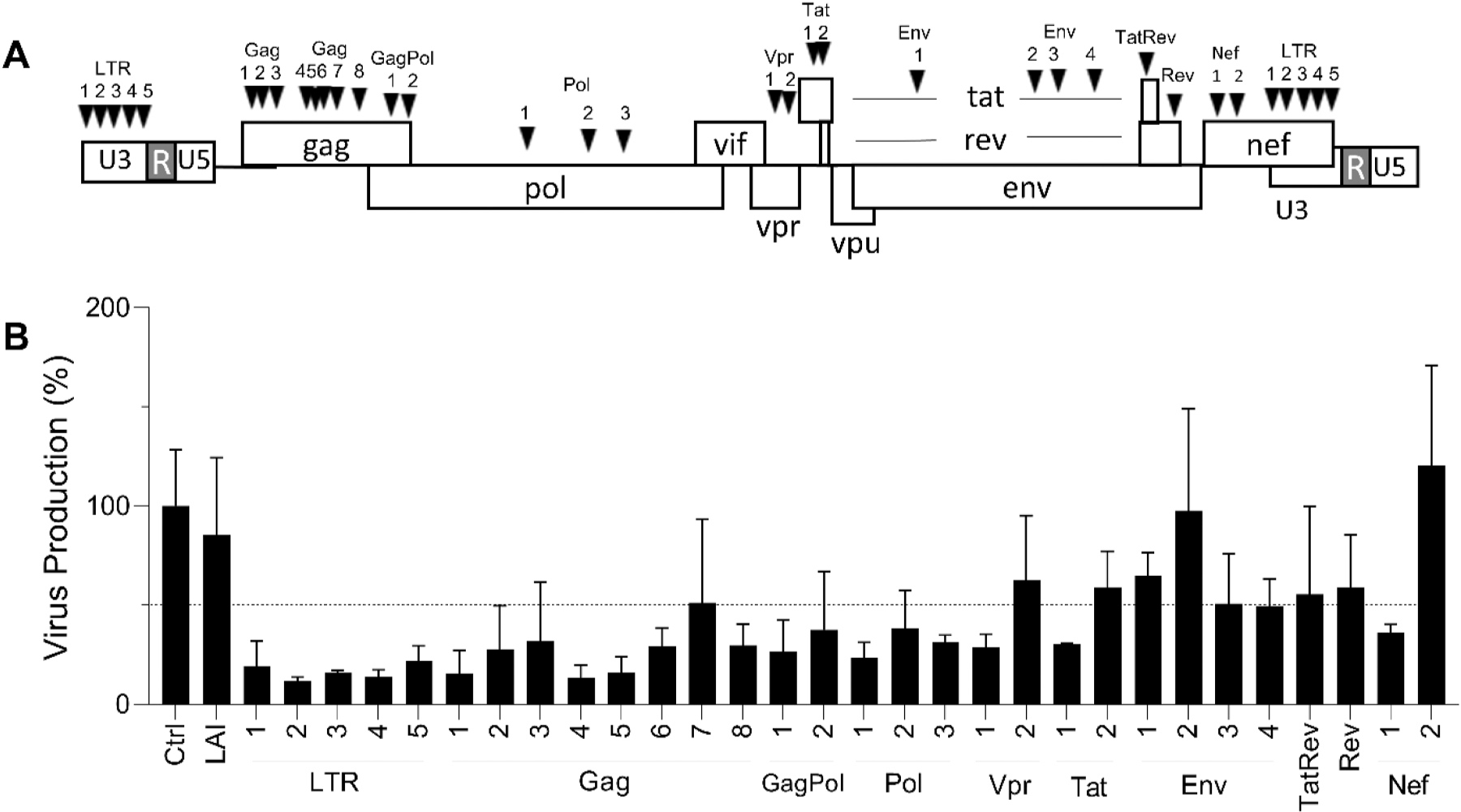
Targeting HIV DNA with Cas 12b and a single gRNA. (A) Thirty gRNAs were designed against the HIV LAI isolate, all targeting highly conserved viral sequences. (B) The anti-HIV activity of the gRNA constructs was tested in transient transfections. Plasmids encoding HIV LAI and Cas12b/gRNA were co-transfected into HEK293T cells. Two days post-transfection, the culture supernatant was collected for CA-p24 ELISA to detect virus production in the supernatant. The CA-p24 level was set at 100% virus production in the control transfection (Ctrl). The dashed line reflects 50% knockdown based on the CA-p24 value of the control Ctrl sample. The data represent the mean ± standard deviation (SD) of three experiments in duplicate.

We therefore decided to design novel ATTN-using anti-HIV gRNA molecules of 23-nt with the CRISPR RGEN tool Software Cas-designer (Fig. 1A, the gRNA sequences are listed in Supplementary Table S1). We designed gRNAs with a perfect match to the genome of the primary virus isolate LAI that we use in our experiments. These gRNAs are rigorously screened to ensure that they do not exhibit any complementarity to the human genome, with a maximum tolerance of 2 mismatches and no bulge [18]. Note that the mismatch tolerance of Cas12b is reduced compared to spCas9, resulting in fewer predicted off-target sites per gRNA and most likely a lower CRISPR-Cas-induced off-target effect compared to spCas9 [35–37]. We selected gRNAs that target relatively conserved HIV sequences to broaden the therapeutic potential towards other HIV isolates and distinct HIV subtypes [38], but also to restrict the likelihood of viral escape as less sequence variation is usually allowed in conserved HIV domains [39]. The gRNAs LTR1 to LTR5 target the two long terminal repeats (LTRs) that flank the viral genome. The 5′LTR copy acts as the HIV transcriptional promoter and other regulatory sequences and the 3′LTR partially overlaps with the nef open reading frame (ORF). All five gRNAs target the U3 domain of the LTR promoter. Of note, any LTR-targeting gRNA will cleave HIV DNA at two positions, thus potentially triggering the excision of a large internal segment with all protein-coding capacity [40]. Such an excision event is considered a robust, safe and thus ideal HIV-inactivation strategy. The other gRNAs (Gag1-8, GagPol1-2, Pol1-3, Vpr1-2, Tat1-2, Env1-4, TatRev, Rev and Nef1-2) target highly conserved HIV sequences that encode the indicated viral proteins. The GagPol gRNA targets two overlapping ORFs (Gag and Pol) and the TatRev gRNA even three (Tat, Rev and Env), thereby increasing the potential impact of Cas12b-editing on HIV fitness.

We first measured the anti-HIV effect in transient assays in which HIV LAI DNA (300 ng) is co-transfected in HEK293T cells with plasmids encoding the Cas12b endonuclease and a single gRNA. We include a Renilla plasmid to control for variation in transfection efficiency and a control gRNA (Ctrl) that targets neither HIV nor the cellular genome. Virus production was measured 2 days after transfection by quantitation of the amount of HIV CA-p24 protein in the culture supernatant (Fig. 1B). Virus production with the Ctrl gRNA was set at 100% and a transfection with just the LAI plasmid served as another control. We marked the 50% knockdown level in Fig. 1B. Most of the designed gRNAs exhibited anti-HIV activity, with Env2 and Nef2 as notable exceptions. The superior activity of the LTR-targeting gRNAs may relate to the editing of two HIV target sites. We selected two LTRs and six “internal” targets to investigate the impact on a spreading virus infection, making a total of eight gRNAs for tests in stably transfected T cells: LTR2, LTR4, Gag5, Pol1, Vpr1, Tat1, TatRev and Nef1.

### 3.2. Cas12b-mediated suppression of HIV replication in gRNA-transduced T cells

**Fig. 2.**
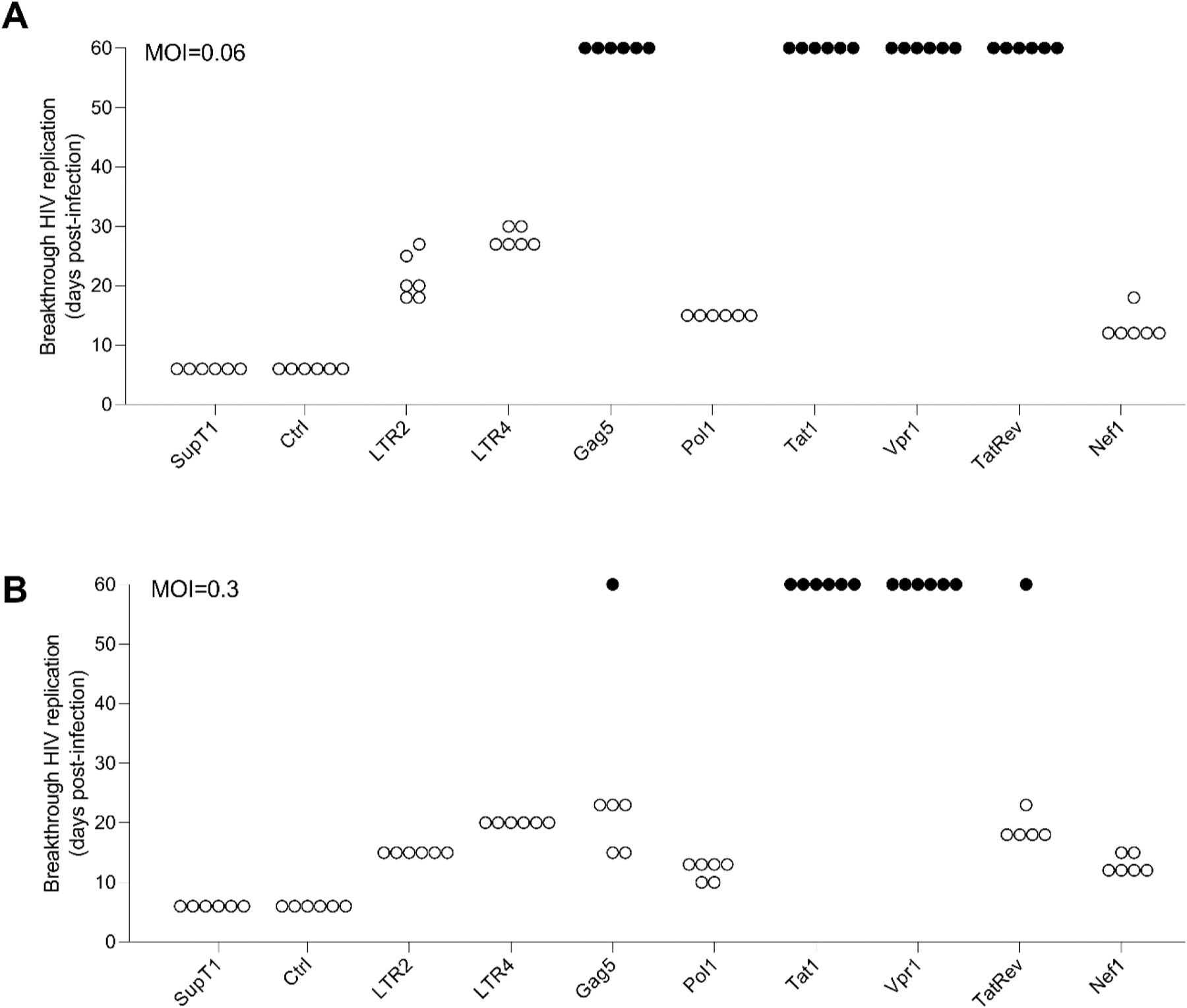
Cas12b-mediated HIV inhibition in long-term T cell cultures. The SupT1 T cell line was stably transduced with lentiviral vectors encoding Cas12b and the indicated gRNAs. Six parallel cultures per gRNA were infected at day 0 with HIV, using an MOI of (A) 0.06 or (B) 0.3 and cultured for 60 days. The day at which massive virus-induced syncytia and cell death was observed is plotted (open circles). Filled circles represent cultures in which HIV was potentially cured as no virus replication was apparent up to day 60. SupT1, non-transduced cells; Ctrl, cells transduced with a control gRNA that does not target the HIV genome.

We stably transduced the T cell line SupT1 with lentiviral vectors that encode both CRISPR components (the Cas12b endonuclease and a single gRNA). For each gRNA-expressing cell line, we infected six parallel cultures with HIV LAI at a multiplicity of infection (MOI) of 0.06 and the appearance of virus-induced syncytia was monitored up to 60 days. We scored unhindered HIV replication for unprotected SupT1 cells and those transduced with the Ctrl gRNA, yielding virus-induced cytopathic effect (syncytia) and cell death at day 6 in all six independent cultures (Fig. 2A, open circles). The cultures transduced with LTR2, LTR4, Pol1, and Nef1 gRNAs showed a weak antiviral effect as delayed virus replication was visible as cytopathic effects and cell death between days 10–30. These initial results indicate that these gRNAs have limited antiviral activity.

More pronounced HIV suppression was observed in cells transduced with Gag5, Tat1, Vpr1 or TatRev, for which all six parallel cultures exhibited permanent HIV suppression up to the end of the experiment at day 60 (Fig. 2A, filled circles). The cultures that appear to show an effective HIV-cure represent the first examples of durable Cas12b-mediated HIV inactivation. To select the most potent inhibitors, we challenged these cells with HIV at a five-fold higher MOI of 0.3 (Fig. 2B). Only the Tat1 and Vpr1 cultures did maintain a robust and permanent HIV block in all 6 cultures. Thus, Tat1 and Vpr1 were identified as the most potent gRNA inhibitors in combination with the Cas12b endonuclease.

### 3.3. HIV escape from Cas12b-pressure in T cell cultures

**Fig. 3.**
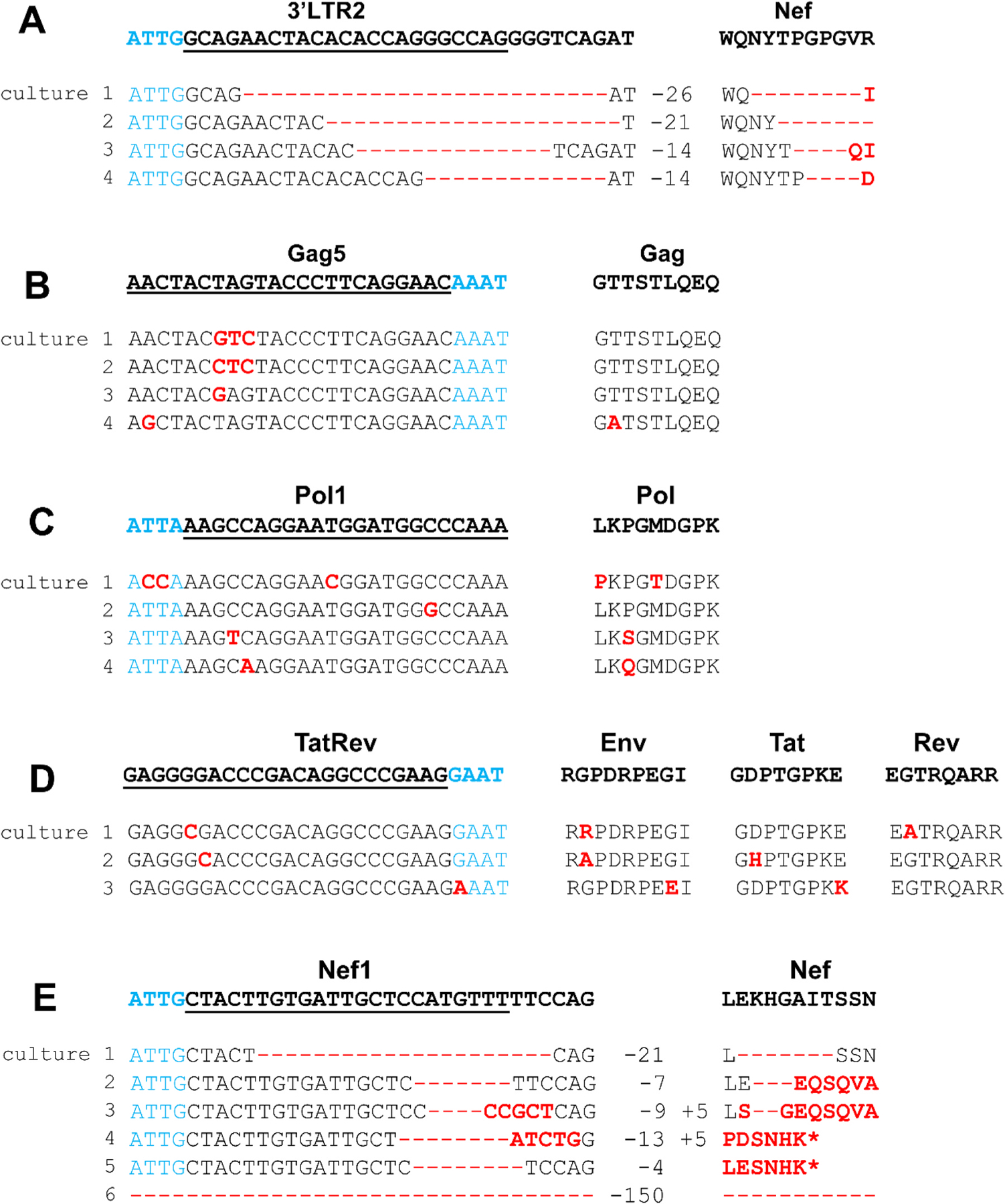
Genome analysis of HIV variants that escape from Cas12b-pressure. (A) LTR2, (B) Gag5, (C) Pol1, (D) TatRev and (E) Nef gRNA guides Cas12b to the respective regions of HIV DNA as shown in Fig. 1A. Candidate escape viruses from cultures in which HIV replication was observed were passaged on gRNA-transduced cells and the cellular DNA was harvested. The gRNA targets in the HIV genome were PCR-amplified and Sanger sequenced and sequences were aligned with the input HIV LAI sequence (wild type or WT on top). The culture number is indicated. The gRNA targets are underlined, the PAM is marked in blue, and the actual cleavage site is indicated with a triangle. Base substitutions are highlighted in red. The size of base deletions (−) is indicated on the right of the sequence. The encoded HIV protein and the amino acids are indicated in the right-hand panels, for TatRev we show the 3 proteins that are translated from the 3 overlapping open reading frames (ORFs). Substitutions are highlighted in red, deletions are indicated by -, *marks stop codons due to a frameshift.

We first analyzed the infected T cell cultures where viral escape from Cas12b pressure may have occurred, for which we focused on the high MOI infections (MOI of 0.3 in Fig. 2B). As a phenotypic test for gRNA-resistance, we passaged the virus-containing supernatant at the peak of infection onto a new batch of gRNA-transduced cells. HIV replication was apparent in several cultures, thus confirming the escape phenotype. To identify the molecular reason for HIV escape we isolated the cellular DNA and PCR-amplified the relevant Cas12b-targets of the integrated HIV DNA genomes. These PCR products were subsequently sequenced and the mutations acquired in the Cas12b targets are plotted in Fig. 3 (4x LTR2 cultures, 4x Gag5, 4x Pol1, 3x TatRev and 6x Nef1). As previously reported for Cas12a-induced lesions, pure insertions were not observed upon Cas12b-editing [22]. Instead, we observed regular deletions and typical Cas12-[18]products we termed “delins” that combine a deletion with a small sequence insertion [22] (Fig. 3E, cultures 3 and 4).

The LTR2 and Nef1 cases that display typical Cas12b-induced deletions in the HIV target will be discussed first (Fig. 3A and E). HIV is apparently capable of replication without these sequences. Although the selected deletions in the LTR remain fairly small, we observed two relatively large in-frame deletions in Nef (21 and 150 bp) and actually also deletions and delins that cause a frameshift in the Nef ORF, thus effectively blocking translation of the C-terminal half of this accessory protein (cultures 2–5). Although Nef is required for high-level virus replication in vivo [41–43], this function is dispensable for replication in T cell lines [44–46]. In fact, Nef has a negative effect on HIV replication in transformed T cell lines, which explains its frequent inactivation in previous studies and in this experiment [47,48].

Less dramatic point mutations were selected in the Gag5, Pol1 and TatRev targets of escape viruses (Fig. 3 B–D). This likely indicates that more dramatic mutations in these three critical ORFs are not compatible with HIV replication and thus not selected. All the observed mutations are likely introduced during the error-prone DNA repair process that acts swiftly upon DNA cleavage, as originally reported for Cas9 [27]. Point mutations were observed that change the Cas12b-target and the encoded amino acid, but these substitutions are apparently compatible with virus replication (e.g. Gag5 culture 4 and Pol1 cultures 1, 3 and 4). In other cases, synonymous or silent codon changes were selected under Cas12b-pressure that do not change the encoded amino acid (Gag5 cultures 1, 2 and 3, and Pol1 culture 2), indicating evolutionary pressure to maintain the function of the encoded viral protein. Escape from gRNA-TatRev may be more difficult as the Cas12b-target overlaps three genes: Env, Tat and Rev. Consistent with this more demanding evolutionary setting, we observed only point mutations and no frameshift mutations that actually would destroy all three critical ORFs. In the three escape cultures analyzed, we scored a silent codon change in Rev (cultures 2 and 3) and Tat (culture 1), but not in Env (Fig. 3D). These results may cautiously suggest that it is more difficult for HIV to accommodate amino acid substitutions in the Tat and Rev domains compared to Env. This finding is consistent with the previous finding that this Rev domain is more critical than the respective Tat and Env domains [49,50].

Although most HIV escape cultures revealed a candidate escape virus, some cultures showed a mixed composition of the viral population sequence (6 ×5′LTR2, 2 ×3′LTR2, 6 ×5′LTR4 and 6 ×3′LTR4). This likely indicates that multiple escape variants developed at around the same time. We analyzed the mixed sequence of these cultures by additional means (TA-cloning and sequencing of individual clones) and presented the results in Supplementary Fig. S1. As expected, we witnessed deletions and delins at the site of DNA cleavage.

### 3.4. An HIV cure imposed by Cas12b in T cell cultures

**Fig. 4.**
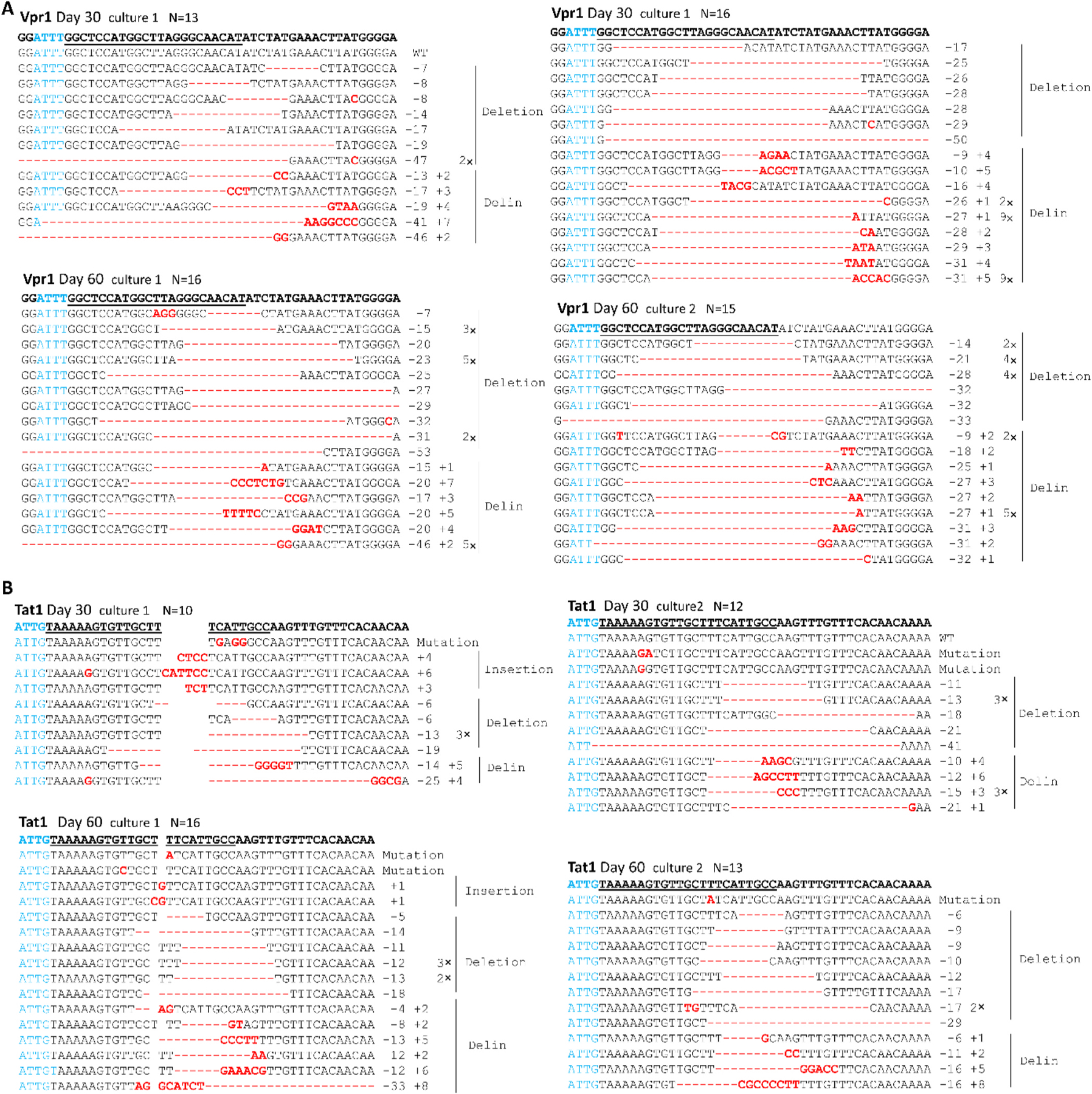
Genome analysis of apparently cured HIV cultures. Cellular DNA was isolated at day 30 and 60 post HIV infection of Cas12b/gRNA-transduced SupT1 T cells. The gRNA-targets in the HIV genome were PCR-amplified (using primers listed in Supplementary Table S2), TA-cloned and Sanger sequenced. The sequencing reads were aligned with the WT HIV sequence shown on top. The gRNA targets are underlined and the PAM is marked in blue. Sequences were grouped according to their class: substitution, pure deletion or insertion and the delin class (shown in red). Behind each sequence, we listed the frequency and the size of the actual deletion (−) and insertion (+). The number of sequences we detected are shown on top. In some cultures no unedited WT sequences were detected. Sequences were analyzed for two gVpr-transduced SupT1 T cultures (A) and two gTat1-cultures (B).

We observed durable HIV suppression up to day 60 in all six-cell cultures equipped with the individual gRNAs Vpr1 and Tat1, and for only one-in-six cultures expressing Gag5 or TatRev (Fig. 2B). To document complete HIV inactivation in these cultures, we performed two additional assays. First, we performed an ultra-sensitive virus detection experiment by addition of unmodified SupT1 cells to a sample of the original cultures taken at day 15, 30 and 60 post-infection. Whereas virus was readily detected in all early samples, the subsequent samples demonstrated a total loss of replication-competent virus (Table 1). Thus, a complete HIV inactivation or cure seems to have been achieved in these cases. Second, we selected representative cultures for a genetic analysis of the HIV genome, where we expect to find lesions introduced by Cas12b-editing that could explain the cure phenotype. The Cas12b targets were PCR-amplified, TA-cloned and sequenced for the day 30 and 60 samples (Fig. 4 and S2). Among the 111 HIV sequences, we observed very few unedited WT genomes at day 30 (1x for Vpr1 culture 1, 1x for Tat1 culture 2) and none at day 60 (Fig. 4), which demonstrates ongoing editing until all target sequences have been modified, concomitant with HIV-inactivation.

We observed a diversity of genetic alterations in the Cas12b target sites: regular deletions (46.9%), delins (45.1%), point mutations (3.7%) and regular insertions (3.3%). Most of these mutations (75.9%) cause a frameshift in the targeted ORFs that are critical for HIV replication, providing compelling evidence for complete HIV sterilization by the Cas12b attack. A seed sequence (5 bases downstream PAM) of the Cas12b system has been described that must be strictly complementary to the guide RNA [18]. The introduced mutations did in most cases not disrupt the PAM and seed sequence (82.0% of the sequences, see Fig. 4, ATTN in blue). This means that an additional round of DNA cleavage and repair would in theory have been possible.

### 3.5. Mutational profile induced by Cas12b-mediated DNA cleavage and subsequent repair

**Fig. 5.**
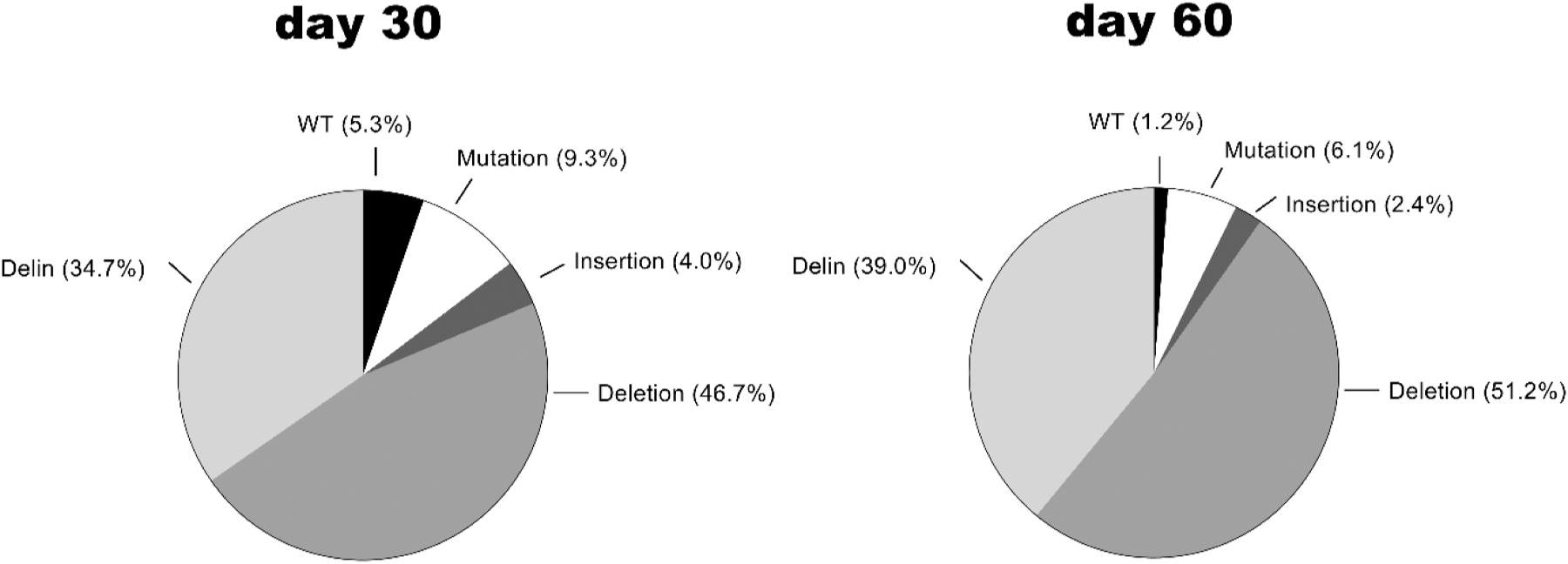
The mutational profile caused by Cas12b editing of HIV DNA. The frequency of unedited HIV target sequences and different types of Cas12b-mediated scars (mutations, insertion, deletion and delin) at day 30 (left) and day 60 (right) is plotted. We analyzed a total of 75 and 82 sequences for the early and late samples, respectively. These sequences are shown in Fig. 4 and Supplementary Fig. S2.

We next calculated the frequency of the different types of genetic lesions introduced by Cas12b of all cured cultures at day 30 and 60 (Fig. 5). First, we observed a dramatic loss of WT HIV sequences, which were reduced to 5.3% after 30 days and further down to 1.2% at day 60 of the CRISPR-treatment. The decline over time may indicate repeated Cas12b-recognition and cleavage of minimally or point-mutated targets that maintain an intact PAM, a phenomenon previously described for Cas12a [22]. Most prominent are the deletions (46.7% and 51.2% at day 30 and 60) and the delins (34.7% and 39.0%, see Fig. 5). We infrequently observed pure sequence insertions (4.0% in early samples and 2.4% in late samples) and HIV sequences with a single point mutation (9.3%) or multiple point mutations (6.1%).

**Fig. 6.**
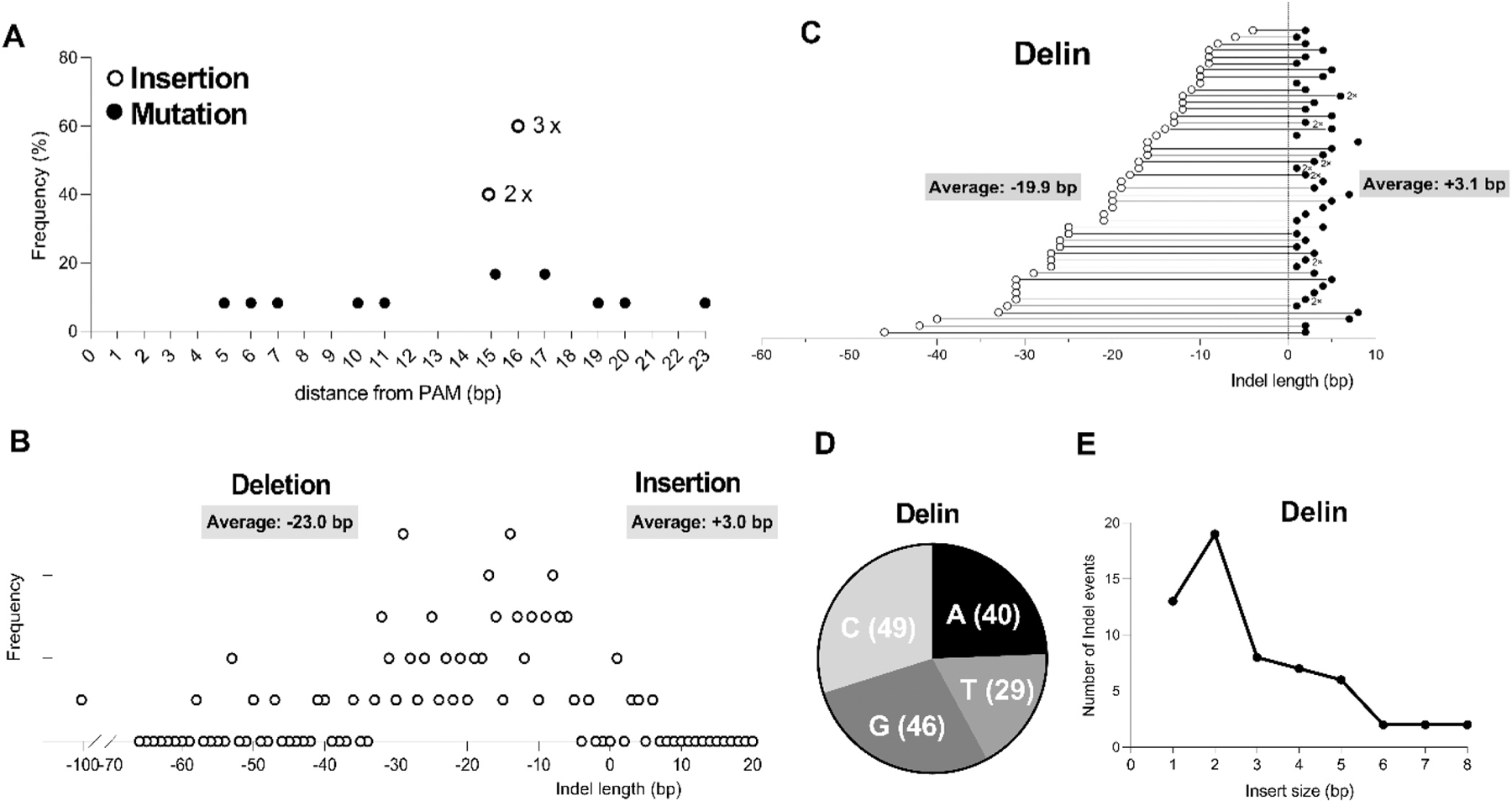
Detailed characteristics of Cas12b-induced indels and delins. (A) Frequency distribution of the distance (in nucleotides, bp) between the mutation and the PAM motif was plotted for insertions (open circles) and mutations (filled circles). (B) Frequency distribution of the size of pure deletions and insertions (collectively termed indels) induced by Cas12b. Deletions are shown in the left panel and insertions in the right panel. The average sizes of the deletions and insertions are indicated. (C) Analysis of the Cas12b-induced delins with the coupled deletion (left, open circles) and insertion (right, filled circles). The respective sizes (in bp) are plotted on the Y-axis. The average sizes of the deletion and insertion components were calculated for all plotted data points. Different clones with the same sizes of the deletion-insertion are marked (e.g. 2x). (D) Nucleotide composition of the insertion-component of delins. This analysis reflects the sense strand of the HIV DNA targets. (E) Frequency distribution of the size of the insertion-component (in bp) of the delin class of mutants.

We next investigated the position at which the mutations occur. Mutations induced by Cas12b are distributed between position 5–23 bp distal of the PAM, with a modestly higher frequency at position 15 and 17 (Fig. 6A). All insertions occurred either at position 15 [3x] or 16 [2x] distal from PAM. Altogether, these results may indicate that the Cas12b nuclease (BhCas12b) cleaves the complementary DNA strand 15–17 bp upstream of the PAM. We plotted the size of the pure deletions and insertions introduced by Cas12b in Fig. 6B, using edited HIV sequences from all cured cultures. The Cas12b products exhibit an average deletion length of 23.0 bp, which is similar to Cas12a (24.3 bp [22]), while the average insertion length is 3.0 bp for Cas12b, while no insertions were observed for Cas12a (Fig. 6B). We also analyzed the delin class of mutations that combine a deletion and small insertion (Fig. 6C). The deletion-component varies in size with an average of 19.9 bp, the coupled insertion has an average size of 3.1 bp. These size trends are roughly similar to that of separate deletions and insertions of regular indel mutations as shown in Fig. 6B. We next assessed whether the insertion component from the delin class exhibits a particular preference for one of the four nucleotides (Fig. 6D). All four bases are frequently present in those inserts, perhaps with a slight preference for G and C. We also show the frequency of the insert sizes in Fig. 6E, and plotted them differently in Fig. 6C, right panel. Insert sizes vary from 1 to 8 bp, with a preference for the smaller 1 and 2 bp inserts.

### 3.6. Improving Cas12b mediated HIV-inhibition with dual gRNAs

**Fig. 7.**
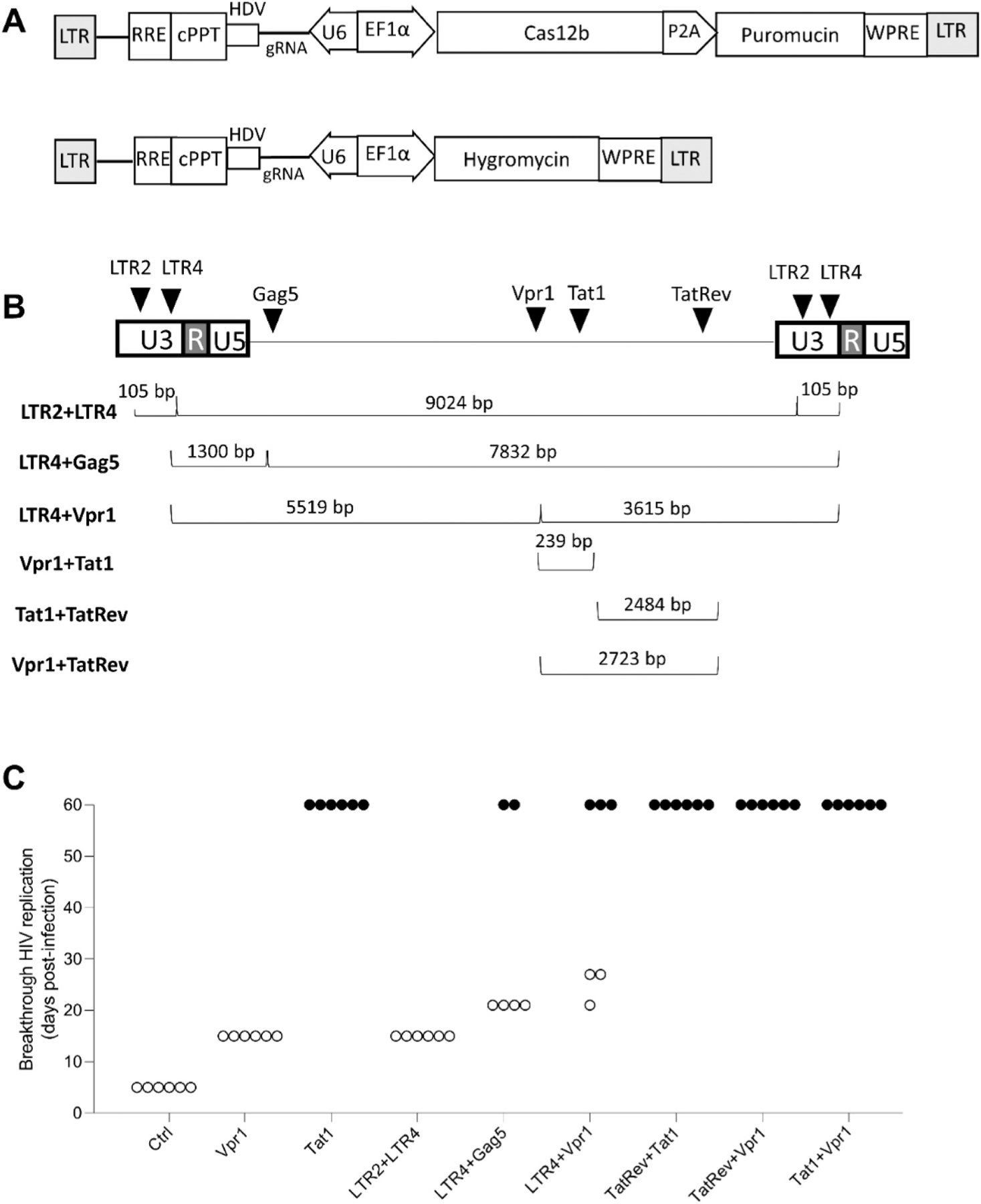
Dual Cas12b attack on HIV DNA. (A) Schematic of the lentiviral constructs encoding Cas12b and gRNA1-HDV and gRNA2-HDV. (B) Schematic of six dual gRNA combinations to target the HIV DNA genome, with the distance between the dual targets indicated in base pairs (bp). The position of the gRNA target sites on HIV DNA is indicated by black triangles. (C) Single versus dual Cas12b-mediated HIV inhibition. Six parallels cultures per single or dual gRNA set were infected with HIV (MOI 0.6) and cultured for 60 days. The day at which massive virus-induced syncytia and cell death was observed was scored (open circles). Candidate cured cultures did not show any sign of virus replication up to day 60 (filled circles). SupT1, non-transduced cells; Ctrl, cells transduced with a control gRNA that does not target the HIV genome.

To test whether the current Cas12b antiviral strategy can be improved, we set out to combine two gRNAs for a dual attack on the HIV DNA genome. A potential additional advantage of a dual attack is that the intervening HIV genome fragment may be excised, yielding more robust HIV-inactivation that will increase the safety of a HIV-cure strategy. We tested seven dual gRNA combinations (Fig. 7). A schematic of the lentiviral vectors carrying Cas12b and a gRNAs with HDV and used to transduce SupT1 cells is shown in Fig. 7A. The different dual gRNA combinations tested are shown in Fig. 7B. Of note, the LTR2 +LTR4 combination targets the HIV proviral DNA genome at four positions, with two targets near the 5′ and 3′ ends, which may trigger efficient excision of the nearly complete integrated provirus. LTR4 was also combined with Gag5 or Vpr1, cleaving the HIV provirus at three sites. TatRev was combined with Tat1 or Vpr1 and Tat1 was combined with Vpr1, the latter combinations can edit HIV DNA at two positions.

We tested the dual gRNA combinations in stably transduced SupT1 cells and included two single gRNA attacks for comparison (Vpr1 and Tat1). For each therapeutic cocktail, 6 independent cell cultures were infected with HIV LAI at an MOI of 0.6 and we monitored virus replication for 60 days (Fig. 7C). Based on the appearance of virus-induced syncytia at day 5, we scored unhindered HIV replication for the 6 control cultures (Ctrl). The level of HIV inhibition by the control single gRNA treatment varied: Vpr1 initially suppressed HIV, but virus escape occurred over time (open circles), whereas Tat1 demonstrated durable HIV suppression up to the end of the experiment in all six cultures (filled circles). For the dual gRNA treatments, we observed HIV breakthrough for LTR2 + LTR4 (6 of 6 cultures), LTR4 + Gag5 (4 of 6) and LTR4 + Vpr1 (3 of 6). We observed robust and durable HIV suppression (6 of 6 cultures) for TatRev +Tat1, TatRev +Vpr1 and Tat1 +Vpr1. Overall, the dual gRNA combinations exhibited more potent HIV inhibition than the single gRNA treatments.

**Fig. 8.**
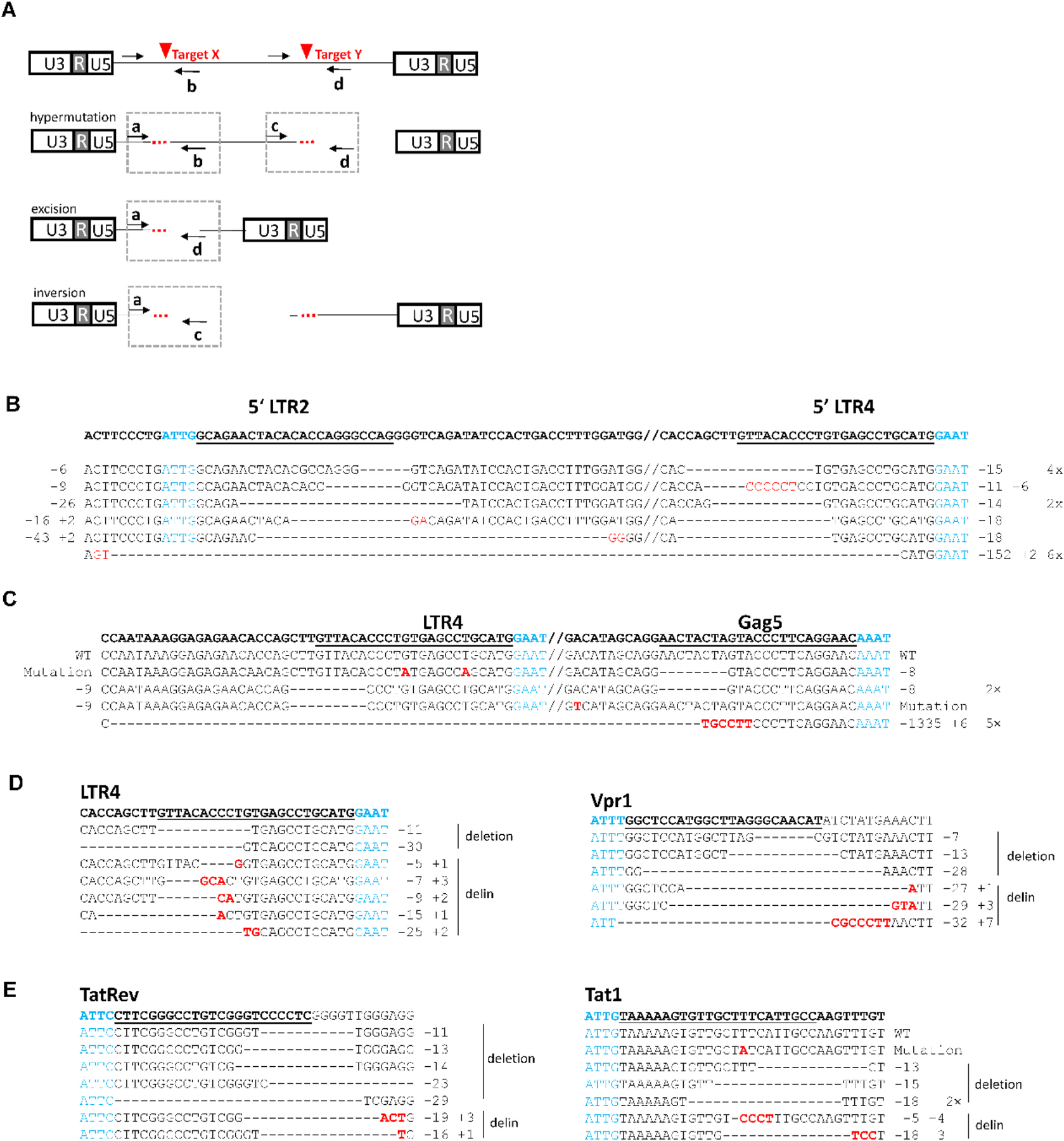
Mutational profile in cultures upon dual CRISPR attack. (A) Schematic of HIV DNA with the position of the gRNA target sites indicated by red triangles and the position of PCR primers a-d by black arrows. The primer combination a + d was used to amplify full-length fragments with either a wild type or mutated sequence. If the distance between cleavage sites is bigger than 1500 bp, each target site is amplified separately using primers a + b and c + d. The excision product and the inversion product will be detected with primers a + d and a + c, respectively. (B-E) Sequence analysis of HIV proviral DNA from one representative culture of transduced SupT1 cells: (B) LTR2 +LTR4, (C) LTR4 +Gag5, (E) LTR4 +Vpr1 and (D) TatRev +Tat1. The cell genome of these cultures was extracted and relevant HIV sequences were subsequently PCR-amplified, TA-cloned and Sanger sequenced. A variety of distinct indels underscore the cure phenotype. See the text for further details.

To analyze the mutational profile of HIV inactivation for some of the “cured” cultures, we PCR-amplified, TA-cloned and Sanger-sequenced the Cas12a targets (Fig. 8). Note that dual Cas12b attack can generate different products, e.g. with mutations at both gRNA target sites (“hypermutation”), excision of the HIV fragment between the two targets or even inversion of that fragment [51–53]. We used a series of primers to distinguish these three products (Fig. 8A). Both targets were frequently edited (Fig. 8B–E), thus favoring the hypermutation scenario over the excision route. We detected only small excision events of LTR fragments that likely are not essential for efficient HIV-1 gene expression and replication in SupT1 T cells (Fig. 8B) [54–56]. Excision was also observed in a single LTR4 +Gag5 culture (Fig. 8C). In general, Cas12b editing was very effective as only a single WT sequence with two unedited targets was left for the LTR4 +Gag5 combination. Concerning the type of lesion introduced, we frequently scored deletions and delins and no pure sequence inserts and inversions were detected. This result is consistent with those presented for Cas12b in Fig. 4 and in the literature for other CRISPR systems [22,26].

### 3.7. Absence of off-target effects induced by CRISPR-Cas12b

**Fig. 9.**
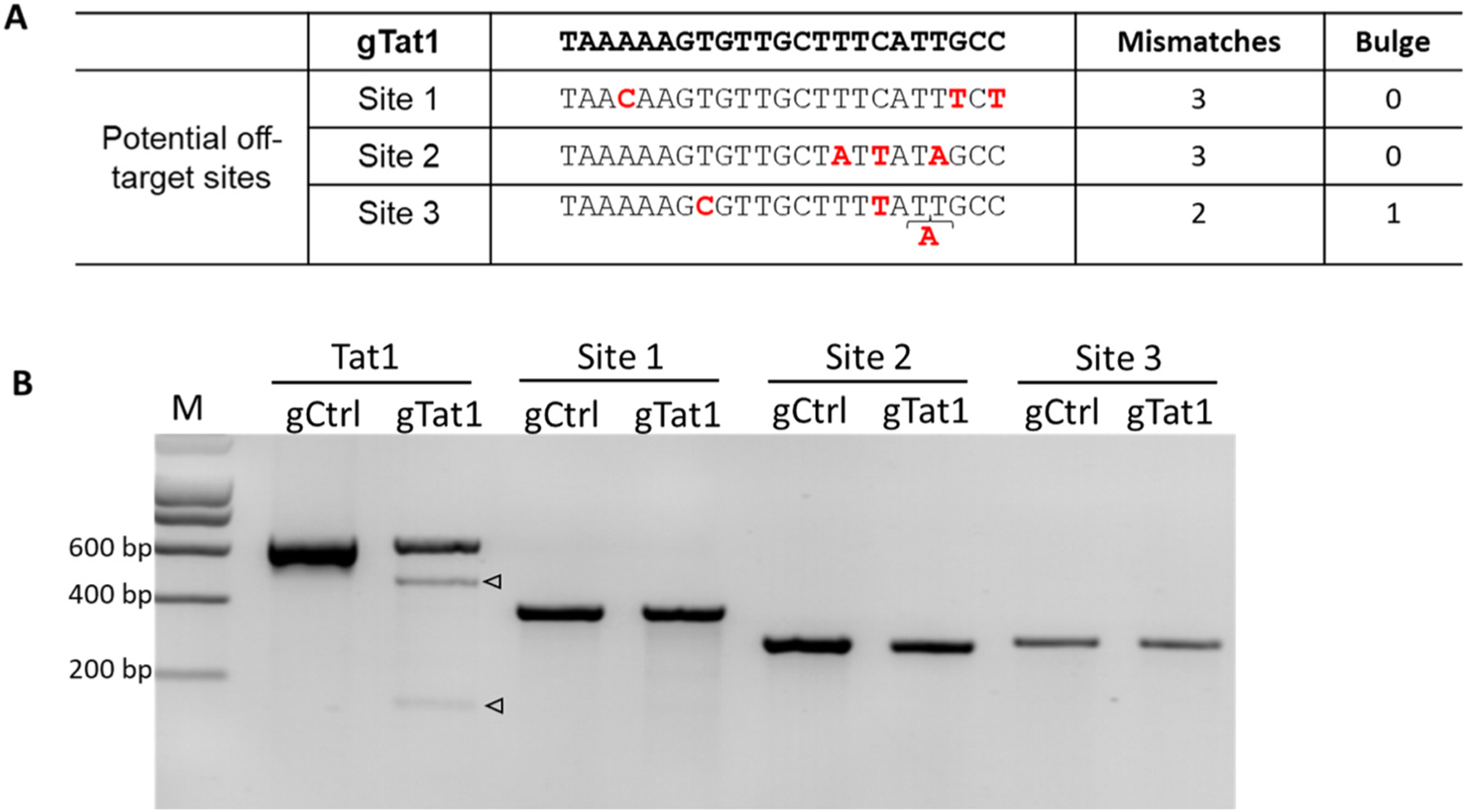
Detection of CRISPR-Cas12b-induced off-target effects. (A) Potential off-target loci with 2 mismatches and a single-base bulge or 3 mismatches and no bulge. Mismatches and bulges are colored in red. (B) T7EI-cleaved bands are indicated with triangles. The M lane contains a DNA ladder. gCtrl is the sample from gCtrl-transduced cells, and gTat1 is from gTat1-transduced HIV-infected cells.

We used the CRISPR RGEN tool and the human genome (Homo sapiens GRCh38/hg38) as template to predict putative off-target loci for the top gRNA candidates. For the four most potent gRNAs (Gag5, Vpr1, Tat1, and TatRev), we did not detect any off-target sites with full complementarity or with a single mismatch. It has been described that off-target effects rarely occur with 2 or more mismatches between gRNA and target DNA for CRISPR-Cas12b-related systems [18]. To verify this, we selected several predicted off-target loci for gTat1 with 2 mismatches plus a single-base bulge or 3 mismatches (without bulge) for further tests (Fig. 9A). To quantify genomic DNA cleavage in SupT1 cells, we used the T7E1 mutation detection assay. To test on-target CRISPR-Cas12b activity, genomic DNA was harvested from gTat1-transduced HIV-infected cells at day 60 post-infection and a 603 bp region around the gTat1 target was PCR-amplified. Due to the death of non-protected gCtrl-Supt1 within a week after HIV infection (Fig. 7C), we used plasmid HIV DNA treated with CRISPR-Cas12b-gCtrl as a negative on-target control for our experiments. The DNA product was denatured and reannealed by heating and cooling, incubated with the T7E1 enzyme, and analyzed on an agarose gel (Fig. 9B). We observed a full-length amplicon in HIV DNA treated with gCtrl in the Tat1 target site, while predictable excision bands (145 bp and 458 bp) were present for gTat1-treated cells. For the three putative gTat1 off-target loci, we used either non-infected gCtrl-transduced SupT1 cells or gTat1-transduced HIV-infected cells. We observed only the full-length amplicon, indicating that no off-targeting occurred.

## 4. Discussion

In the gene therapy field, zinc finger endonucleases (ZFNs) and transcription activator-like effector nucleases (TALENs) were initially developed for gene modification and these tools were subsequently used in attempts to inactivate the integrated HIV proviral DNA [57–59]. Recent studies indicated that CRISPR-Cas mediated genome editing is more efficient and probably safer [60–62]. CRISPR-Cas9 was also applied for inhibition of HIV replication, but achieving a full cure in a T cell line required a combination of two gRNAs [23,26]. We next demonstrated that Cas12a can sterilize all infectious HIV with only a single gRNA, presenting a highly effective and alternative HIV cure approach [22]. However, one of the remaining issues concerns the efficiency of gene delivery to the cells that harbor an integrated HIV provirus. A true in vivo cure would likely require the transduction of most or even all of these reservoir cells. This requires a high transduction titer of the vector used, in our case the lentiviral vector, which is significantly reduced by incorporation of the sizeable CRISPR system. For this reason, smaller CRISPR systems need to be discovered or engineered. The discovery and optimization of the smaller Cas12b system gets us closer to this eventual clinical goal [18]. In this study, Cas12b was shown to have similar anti-HIV potency as Cas12a, but the significantly smaller size of the endonuclease would allow the production of lentiviral vectors with a higher titer.

We previously suggested that distinct DNA breaks introduced by different Cas endonucleases trigger the assembly of diverse DNA repair complexes that lead to distinct mutations or lesions in the HIV DNA [22]. In general, Cas12b induced a similar indel pattern as Cas12a with 51.2% deletions (Cas12b) versus 53.4% (Cas12a) and 39.0% delins (Cas12b) versus 44.4% (Cas12a). Cas12b generated only a very small percentage of insertions (Fig. 6B), which is a frequent editing scar for Cas9 [22,26]. This likely relates to the 7 nt 5′-overhang of the Cas12b cleavage product, which will preferentially recruit exonuclease Artemis during NHEJ repair [63]. Artemis is capable of resecting the overhang that leads to a blunt DNA end, this process also suggests a 7-nt loss prior indel formation during DNA repair. In contrast, a blunt DNA end generated by Cas9 can be ligated instantaneously. Different from Cas12a and Cas12b, the Cas9 endonuclease generated 30.8% pure insertions, 35.8% deletions and 25.0% delins [22]. In terms of base insertion type, Cas12a and Cas12b tend to induce more G and C insertions than A and T (Cas12a: G>C>A>T, Cas12b: C>G>A>T). In terms of insertion size, Cas12a and Cas12b triggered 1 and 2 bp insertions, especially the latter. Overall, we observed a similar mutational profile for Cas12a and Cas12b [64,65]. The notable difference with the Cas9-introduced DNA scars may explain the superior HIV cure results obtained for Cas12a and Cas12b The distinguishable dissimilarity observed in the DNA scars introduced by Cas9 may elucidate the superior outcomes in HIV cure achieved with Cas12a and Cas12b. This can be attributed to the fact that the repair of staggered DNA lesions is more prone to enduring significant sequence loss compared to blunt end lesions [64].

In our previous HIV cure research, we proposed that excision of HIV sequences between two targets would be the ideal HIV editing product because it will prevent the future generation of any infectious virus. However, for excision you need two gRNAs and the introduction of the second gRNA cassette will hamper the packaging efficiency of viral vectors, resulting in a reduced transduction titer. Nevertheless, we tested different dual gRNA combinations for the ability to induce Cas12b-mediated excision (Fig. 7). Overall, we observed more potent HIV inhibition for dual gRNA combinations than the single gRNA treatments, except for Tat1 combinations (Fig. 7). A striking and unexpected result is that the LTR2 +LTR4 combination, which could theoretically induce four DSBs and trigger the excision of most HIV sequences, demonstrated poor antiviral activity and led to HIV breakthrough in all six T cell cultures (Fig. 7C). Moreover, one would perhaps expect the excision of large internal HIV segments by dual gRNA attack (Fig. 8). However, we detected only small excision events and regular deletions and delins were frequently observed in the target sites of cured T cells (Fig. 8). These results demonstrate that most dual gRNA combinations prevent HIV escape and cured the cells of infectious HIV by hypermutation and not by excision (Fig. 8), exactly as we described for Cas12a [22]. We previously indicated that the kinetics of DNA cleavage versus DNA repair likely determine this specific outcome. If the DNA is swiftly repaired after the first cut before the second cut is made, excision is simply avoided.

Although this finding may reduce the attractiveness of Cas12b for HIV-inactivation approaches, this editing system also has some clear advantages. Cas12b outperformed Cas9 in inactivating the HIV genome with a single gRNA [27]. The smaller size of Cas12b compared to other endonucleases could likely facilitate more efficient vector-mediated delivery, which is especially important for future in vivo applications. We observed that the titer of the lentivirus vector was reduced for the larger transgene spCas9 compared to Cas12b (Supplementary Fig. S3). As the number of produced vector particles was not affected, this means that the use of larger transgenes may cause the production of some empty virion particles that lack an RNA genome. Recently developed miniature CRISPR-Cas systems such as Cas12f and Casphi may offer a promising gene-editing solution if their activity is satisfactorily [66,67].

There may be other applications where the unique properties of Cas12b can be exploited. One could think of research strategies where nucleotide insertions are not desired, e.g. to avoid the creation of neo-epitopes in protein-encoding genes. Cas12b may also outperform Cas9 in strategies designed to disrupt gene function as somewhat larger deletions are introduced (Fig. 6). We also compared the off-target effects of our best gRNA candidates on the human genome for the Cas12b, Cas12a and Cas9 endonucleases [65]. Note that the PAM sequence of Cas9 is widely distributed in the human genome and consequently the frequency of off-target sites is higher for Cas9 than Cas12a and Cas12b (Supplementary Table S3). In general, we envisage that Cas12b could become the favorable editing tool to inactivate protein-encoding genes, but the induced off-target effects must be studied in greater detail before the system is ready for clinical application.

## 5. Conclusions

The major challenges for CRISPR-Cas-based curative therapies of HIV lies in finding the “best” combinatorial CRISPR-Cas regimen and in developing a highly efficient and specific targeting strategy to reach all the cell types and tissues that compose the HIV reservoir. We focus on optimization of delivery by the lentiviral vector, especially by selecting the smallest, yet active and safe CRISPR-Cas system. Cas12b can achieve full HIV inactivation with only a single gRNA, although the combined use of two antiviral gRNAs improved the anti-HIV potency and triggered more grossly mutated HIV proviruses. These studies provide proof-of-concept that this approach towards inactivation of integrated HIV genomes may eventually be used to reach a permanent HIV cure.

## Supplementary Material

Table S1. gRNAs targeting HIV-1

CRISPR-Cas systems

Figure S1

Figure S2

Figure S3

## Figures and Tables

**Table 1:** Ultra-sensitive virus rescue assay.

gRNA	Culture	Day15	Day30	Day60
Gag5	1	+	−	−
Vpr1	1	+	−	−
	2	+	−	−
	3	+	−	−
	4	+	−	−
	5	+	−	−
	6	+	−	−
Tat1	1	+	−	−
	2	+	−	−
	3	+	−	−
	4	+	−	−
	5	+	−	−
	6	+	−	−
TatRev	1	+	−	−

## Data Availability

Data will be made available on request.

## Research in context

### Evidence before this study

Clustered regularly interspaced short palindromic repeat (CRISPR)-Cas9-based approaches were used to inactivate integrated HIV DNA in human cells in vitro and in vivo in infected humanized mice. These studies provided proof of concept that the HIV reservoir could be attacked and inactivated by CRISPR-Cas9-based approaches. Despite these impressive results, the large size of Cas9 transgene cassettes impedes implementation in gene therapy applications with viral vectors that have a limited capacity to package cargo, the “packaging capacity”. We propose to minimize the size of the cargo by use of the smaller nuclease Cas12b.

### Added value of this study

The Cas12b member of the CRISPR-Cas type V endonuclease family was recently identified. The added value of the current focus on Cas12b for HIV-inactivation is multi-fold. First, the new Cas12b system has been studied less intensively than Cas9 and its ability to inhibit replication-competent HIV was not probed yet. Second, the Cas12b nuclease has a significantly smaller size (1108 amino acids or aa) than spCas9 and fnCas12a (1368 and 1307 aa, respectively), making it a more suitable tool for gene therapy applications that use a viral delivery system with a limited packaging capacity. Third, Cas12b may be particularly attractive for HIV genome inactivation because it generates relatively large deletions and because it is very sensitive to mismatches between the guide RNA (gRNA) and target DNA, thus causing less off-target effects than Cas9 and Cas12a and possibly presenting a safer system for future clinical applications. Also limited information is available on the type of lesion introduced by the Cas12b endonuclease, which may also affect the efficiency of HIV-inactivation.

### Implications of all the available evidence

This study provided the proof-of-concept for full inactivation of integrated HIV DNA in infected T cells. This is an important result toward an HIV cure strategy and will trigger further investigations in primary cells and animal models and eventually in human clinical trials.
