# Supplementary material for: CRISPR-Cas12b enables a highly efficient attack on HIV proviral DNA in T cell cultures
Source: Biomed Pharmacother. Author manuscript; Available in PMC 2024 Jul 8. (PMC11228593; doi:10.1016/j.biopha.2023.115046)
Supplement: Table S1. gRNAs targeting HIV-1 [file NIHMS1999423-supplement-Table_S1__gRNAs_targeting_HIV-1.docx]

**Table S1. gRNAs targeting HIV-1**

| **gRNA** | **Position in HIV-1 LAI DNA** | **Orientation** | **PAM** | **Sequence (5’-3’)** |
| --- | --- | --- | --- | --- |
| LTR1 | 16-38, 9148-9170 | sense | ATTC | ACTCCCAACGAAGACAAGATATC |
| LTR2 | 83-105, 9215-9237 | sense | ATTG | GCAGAACTACACACCAGGGCCAG |
| LTR3 | 156-178, 9288-9320 | anti-sense | ATTG | GCCTCTTCTACCTTATCTGGCTC |
| LTR4 | 203-225, 9335-9357 | anti-sense | ATTC | CATGCAGGCTCACAGGGTGTAAC |
| LTR5 | 285-307, 9417-9439 | sense | ATTT | CATCACGTGGCCCGAGAGCTGCA |
| Gag1 | 1315-1337 | sense | ATTA | TCAGAAGGAGCCACCCCACAAGA |
| Gag2 | 1341-1363 | sense | ATTT | AAACACCATGCTAAACACAGTGG |
| Gag3 | 1362-1384 | anti-sense | ATTT | GCATGGCTGCTTGATGTCCCCCC |
| Gag4 | 1397-1419 | anti-sense | ATTC | TGCAGCTTCCTCATTGATGGTCT |
| Gag5 | 1503-1525 | anti-sense | ATTT | GTTCCTGAAGGGTACTAGTAGTT |
| Gag6 | 1574-1596 | sense | ATTT | ATAAAAGATGGATAATCCTGGGA |
| Gag7 | 1637-1659 | sense | ATTC | TGGACATAAGACAAGGACCAAAA |
| Gag8 | 1737-1759 | sense | ATTG | GATGACAGAAACCTTGTTGGTCC |
| GagPol1 | 2104-2126 | anti-sense | ATTC | CCTGGCCTTCCCTTGTAGGAAGG |
| GagPol2 | 2300-2322 | anti-sense | ATTG | TGACGAGGGGTCGTTGCCAAAGA |
| Pol1 | 2622-2644 | sense | ATTA | AAGCCAGGAATGGATGGCCCAAA |
| Pol2 | 4194-4216 | anti-sense | ATTT | CCTCCAATTCCTTTGTGTGCTGG |
| Pol3 | 4704-4726 | anti-sense | ATTC | ATAGATTCTACTACTCCTTGACT |
| Vpr1 | 5707-5729 | sense | ATTT | GGCTCCATGGCTTAGGGCAACAT |
| Vpr2 | 5785-5807 | sense | ATTC | TGCAACAACTGCTGTTTATCCAT |
| Tat1 | 5949-5968 | sense | ATTG | TAAAAAGTGTTGCTTTCATTGCC |
| Tat2 | 5967-5989 | sense | ATTG | CCAAGTTTGTTTCACAACAAAAG |
| Env1 | 6623-6645 | sense | ATTA | ACCCCACTCTGTGTTAGTTTAAA |
| Env2 | 6883-6905 | anti-sense | ATTG | GCTCAAAGGATACCTTTGGACAG |
| Env3 | 7421-7443 | anti-sense | ATTA | AACAGTTGTGTTGAATTACAGTA |
| Env4 | 7679-7701 | anti-sense | ATTG | TCCCTCATATCTCCTCCTCCAGG |
| TatRev | 8443-8465 | anti-sense | ATTC | CTTCGGGCCTGTCGGGTCCCCTC |
| Rev | 8511-8533 | sense | ATTC | GATTAGTGAACGGATCCTTAGCA |
| Nef1 | 8958-8980 | anti-sense | ATTG | CTACTTGTGATTGCTCCATGTTT |
| Nef2 | 9053-9075 | anti-sense | ATTG | GTCTTAAAGGTACCTGAGGTGTG |

**Table S2. Primers used for PCR and Sanger sequencing**

| **Primer** | **Oligonucleotide Sequence (5’-3’)** |
| --- | --- |
| 5’LTR_Forward | TGGATCTACCACACACAAGG |
| 5’LTR_Reverse | CTGCTTGCCCATACTATATGTTT |
| 3’LTR_Forward | GAGCAATCACAAGTAGCA |
| 3’LTR_Reverse | TCTGAGGGATCTCTAGTTAC |
| Gag_Forward | ACCTAGAACTTTAAATGCATGG |
| Gag_Reverse | CGGTCTACATAGTCTCTAAAGG |
| Pol_Forward | GTATGATCAGATACTCATAGAA |
| Pol_Reverse | GGCAAATACTGGAGTATTGTA |
| Vpr_Forward | TGCCTAGTGTTACGAAACTGA |
| Vpr_Reverse | GATAGAGAAACTTGATGAGTCTG |
| Nef_Forward | CTATAAGATGGGTGGCAAGT |
| Nef_Reverse | TAGATCCACAGATCAAGGATATCT |
| TatRev_Forward | AATTAGATAAATGGGCAAGTTTGT |
| TatRev_Reverse | AGTTCCACAATCCTCGTTACAA |

|  | **gRNA** | **PAM** | **Sequence (5’-3’)** | **Mismatches** | **Found targets** |
| --- | --- | --- | --- | --- | --- |
| **Cas9** | Gag1 | AGG | GTTAAAAGAGACCATCAATG | 1 | 0 |
|  |  |  |  | **2** | **4** |
|  |  |  |  | **3** | **13** |
|  | Pol3 | AGG | GCATGGGTACCAGCACACAA | 1 | 0 |
|  |  |  |  | **2** | **2** |
|  |  |  |  | **3** | **11** |
|  | TatRev | AGG | TCTCCGCTTCTTCCTGCCAT | 1 | 0 |
|  |  |  |  | **2** | **1** |
|  |  |  |  | **3** | **20** |
|  | Env2 | GGG | GGAGCAGCAGGAAGCACTAT | 1 | 0 |
|  |  |  |  | 2 | 0 |
|  |  |  |  | **3** | **21** |
| **Cas12a** | Gag1 | TTTG | TTCCTGAAGGGTACTAGTAGTTC | 1 | 0 |
|  |  |  |  | 2 | 0 |
|  |  |  |  | **3** | **1** |
|  | Tat1 | TTTA | GGCTGACTTCCTGGATGCTTCCA | 1 | 0 |
|  |  |  |  | 2 | 0 |
|  |  |  |  | 3 | 0 |
|  | Tat2 | TTTA | CAATAGCAAGTGGTACAAGCAGT | 1 | 0 |
|  |  |  |  | 2 | 0 |
|  |  |  |  | 3 | 0 |
|  | TatRev | TTTG | ATAGAGAAACTTGATGAGTCTGA | 1 | 0 |
|  |  |  |  | 2 | 0 |
|  |  |  |  | 3 | 0 |
| **Cas12b** | Gag5 | ATTT | GTTCCTGAAGGGTACTAGTAGTT | 1 | 0 |
|  |  |  |  | 2 | 0 |
|  |  |  |  | 3 | 0 |
|  | Vpr1 | ATTT | GGCTCCATGGCTTAGGGCAACAT | 1 | 0 |
|  |  |  |  | 2 | 0 |
|  |  |  |  | 3 | 0 |
|  | Tat1 | ATTG | TAAAAAGTGTTGCTTTCATTGCC | 1 | 0 |
|  |  |  |  | 2 | 0 |
|  |  |  |  | **3** | **2** |
|  | TatRev | ATTC | CTTCGGGCCTGTCGGGTCCCCTC | 1 | 0 |
|  |  |  |  | 2 | 0 |
|  |  |  |  | 3 | 0 |

**Table S3. Potential off-target sites for Cas9, Cas12a and Cas12b**
