# Supplementary figures and images for: CRISPR-Cas12b enables a highly efficient attack on HIV proviral DNA in T cell cultures
Source: Biomed Pharmacother. Author manuscript; Available in PMC 2024 Jul 8. (PMC11228593; doi:10.1016/j.biopha.2023.115046)

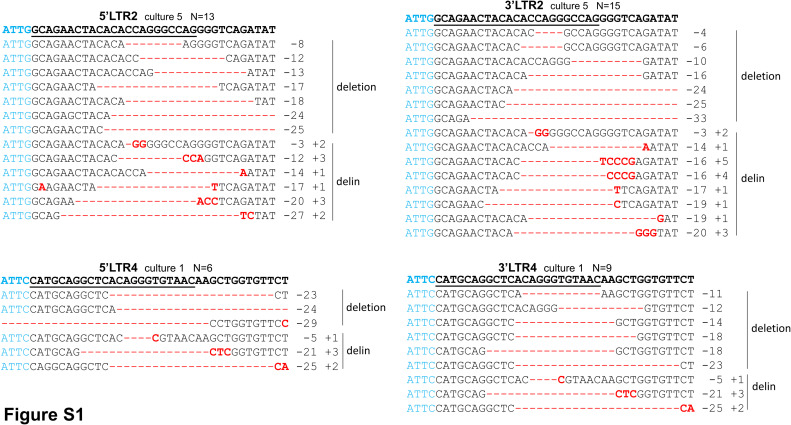

Supplement: Figure S1 [file NIHMS1999423-supplement-Figure_S1.jpg]

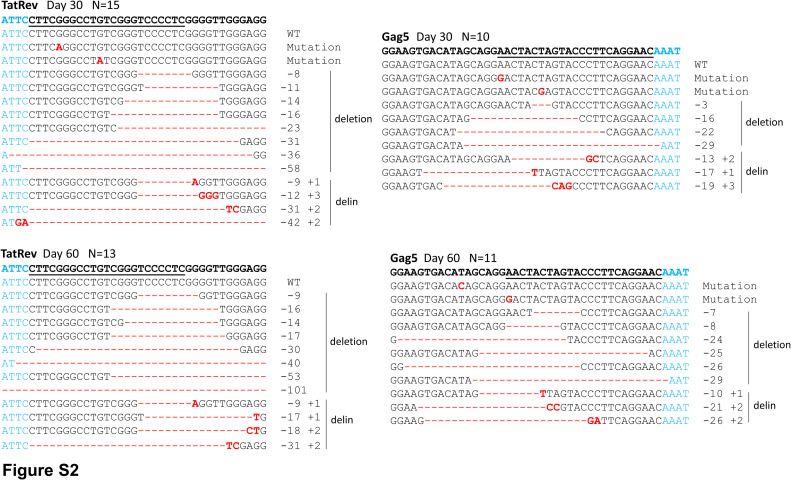

Supplement: Figure S2 [file NIHMS1999423-supplement-Figure_S2.jpg]

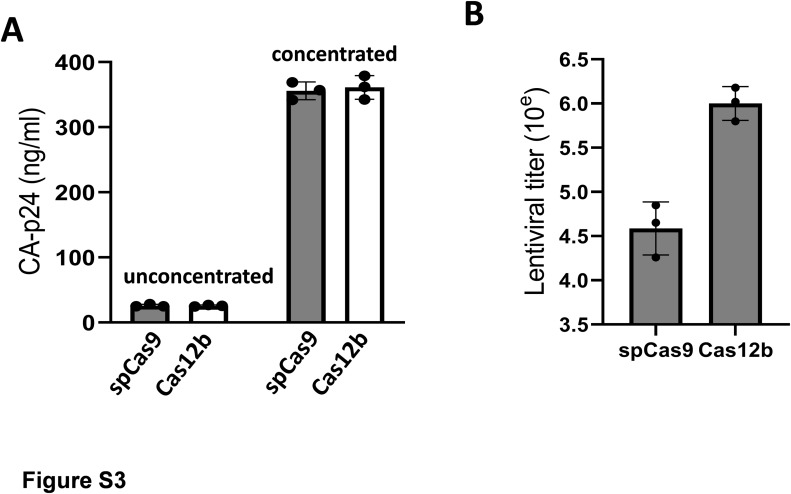

Supplement: Figure S3 [file NIHMS1999423-supplement-Figure_S3.jpg]
